# Supplementary material for: Nanoemulsified Formulation of Cedrela odorata Essential Oil and Its Larvicidal Effect against Spodoptera frugiperda (J.E. Smith)
Source: Molecules. 2022 May 6;27(9):2975. doi: 10.3390/molecules27092975 (PMC9101729; doi:10.3390/molecules27092975)
Supplement: Supplementary file 1 [file molecules-27-02975-s001.zip › molecules-1687530-supplementary.pdf]

Table S1. Essential oils evaluated for the control of *Spodoptera frugiperda*

Family	Plant	Common name	Majority components	Larval stage	Effect	Essential oil concentration	Application	REF
Myrtaceae	Eugenia caryophyllata	Clove	information not available	first instar	anti-feeding	25	ingestion	[1]
Zingiberacea	Curcuma longa	Turmeric	information not available	first instar	anti-feeding	25	ingestion	[1]
Poaceae	Cymbopogon martinii	Palmarosa	information not available	first instar	anti-feeding	25	ingestion	[1]
Poaceae	Cymbopogon winterianu	Java	citronellal (42,15%), citronellol (22,03%) and geran	neonatas	insecticidal, 80%	50	exposition	[2]
Piperaceae	Piper aduncun	Matico	dillapiole	third instar	insecticidal, 50%	1169.7	topical	[3]
Piperaceae	*Piper hispidinervum*		safrole (82%)	first instar	anti-feeding, insecticidal, 50%	16200	ingestion	[4]
Piperaceae	*Piper hispidinervum*		safrole (82%)	third instar	anti-feeding, insecticidal, 50%	9400	ingestion	[4]
Lamiaceae	*Ocimum gratissimum*	albahaca de clavo	(E)-Anethole, Limonene, Eugenol	third instar	insecticidal, 50%	1,52 (1,36–1,67) mg/g inse	topical	[5]
Myrtaceae	*Eucalyptus staigeriana*	corteza de hierro limón	Limonene, Geranial, Neral	third instar	insecticidal, 50%	3,20 (2,41–4,07) mg/g de ir	topical	[5]
Apiaceae	*Foeniculum vulgare*	hinojo	Limonene, (E)-Anethole, a-Pinene	third instar	insecticidal, 50%	5,04 (4,13–5,96) mg/g de ir	topical	[5]
Myrtaceae	Corymbia citriodora	eucalipto limón	information not available	fourth instar	insecticidal, 50%	0.1033 uL/insect	topical	[6]
Myrtaceae	Eucalyptus urograndis	eucalipto rosado	information not available	fourth instar	insecticidal, 50%	0.5424 uL/insect	topical	[6]
Myrtaceae	Eucalyptus urophylla	Timor white gum	information not available	fourth instar	insecticidal, 50%	0..4099 uL/insect	topical	[6]
Lamiaceae	Ocimum gratissimum	clove Basil	p-cymene, γ-terpinene, and thymol	third instar	insecticidal, 50%	0.02 µl/insect and 0.171 µl	topical	[7]
Rapateaceae	*Stegolepis guianensis*		β-myrcene (69.3–79.7%); 2-undecanone (8.37–10.8	fourth instar	insecticidal, 50%	8,09-7.11 µL/mL para cepa	exposition	[8]
Verbenaceae	Lippia origanoides Kunth	romero	Acetophenone (32%), Carvacrol (30%), α-Himachale	third instar	insecticidal, 50%	0,001 % por exposición y 0	exposition	[9]
Poaceae)	Cymbopogon citratus [D	limoncillo	Menthone (56%), γ-Terpinene (18%)	third instar	insecticidal, 50%	0.008% y 1.151% para exp	exposition	[9]
Poaceae	Cymbopogon winterianu	cintronela	3-Carene (29%), (E,E)-2,4-Decadienal (26%), α-Citral	third instar	insecticidal, 50%	0.159% y 1.348% para exp	exposition	[9]
Rutaceae	Citrus aurantium	naranjo amargo	D-limonene (83.3%), sabinene (4.7%), α-thujene (3.3	second instar	anti-feeding, 78-90% mortality	100-250	exposition	[10]
Meliaceae	Azadirachta indica			second instar	insecticidal, 50%, 90%	9500 and 17,230	exposition	[11]

Table S2. Compounds identified in the chromatographic analysis of Neem oil used as positive control in the present work

Peal number	RT	Compound	Percentage (%)
1	17.489	squalene	1.005
2	19.445	palmitic acid	10.24
3	20.475	Oleic acid	5.317
4	20.73	decanoic acid	9.573
5	21.291	Linoleic acid	40.44
6	21.456	stearic acid	21.31
7	23.146	pentadecanoic acid	4.453
		**Total area (%)**	**92.34**

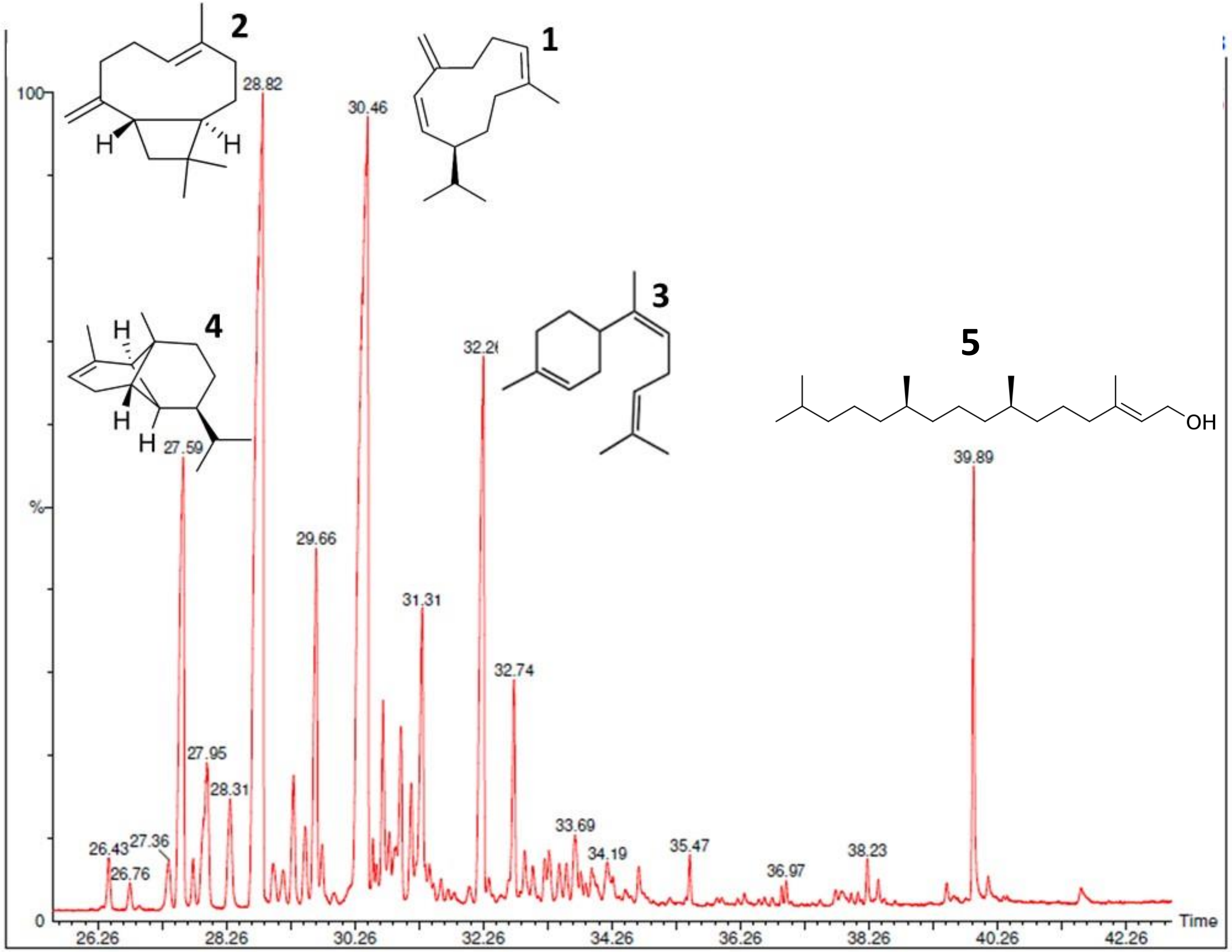

Figure S1. GC-MS Chromatogram of the essential oil extracted from the *Cedrela odorata* dehydrated leaves. Batch 1 collected on July, 2018. Major components: 1 = Germacrene D, 2 = Caryophyllene, 3 = cis-α-Bisabolene, 4 = Copaene, 5=Phytol.

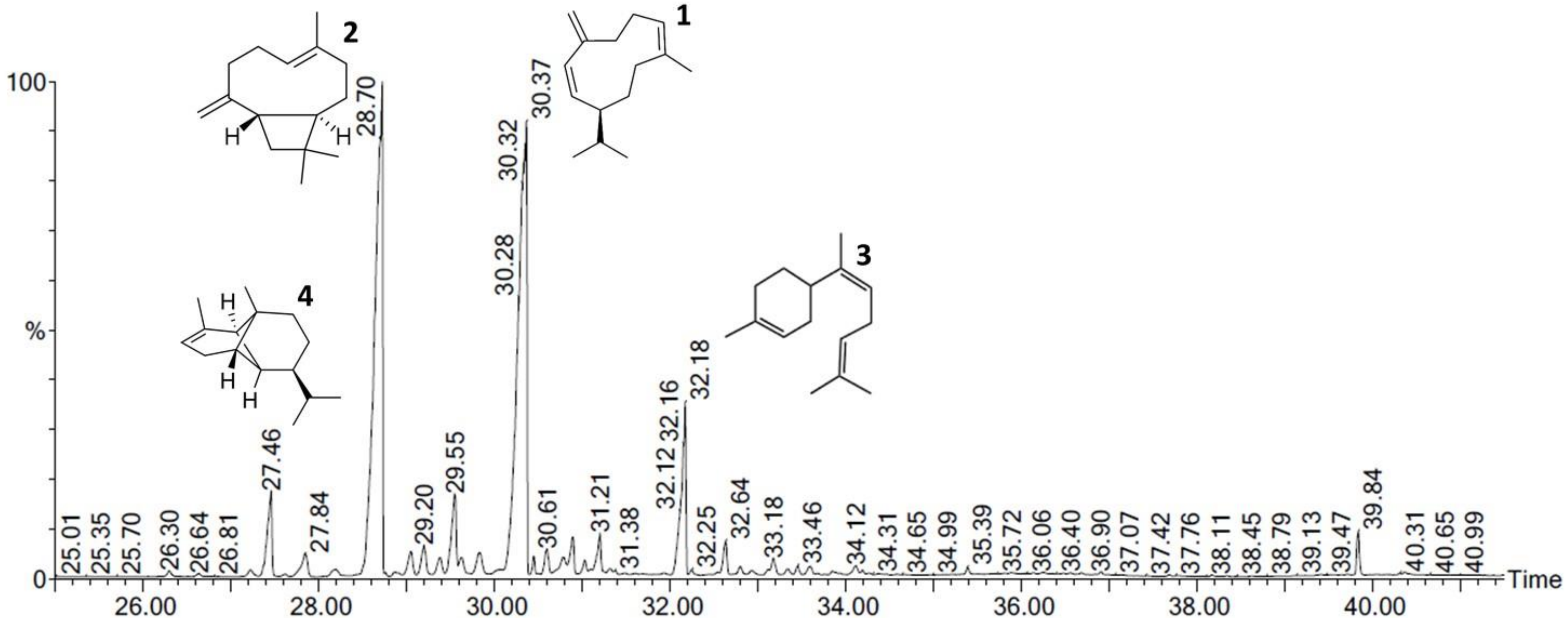

Figure S2. GC-MS Chromatogram of the essential oil extracted from the *Cedrela odorata* fresh leaves. Batch 1 collected on July, 2018. Major components: 1 = germacrene D, 2=caryophyllene, 3= cis-α-bisabolene, 4=copaene.

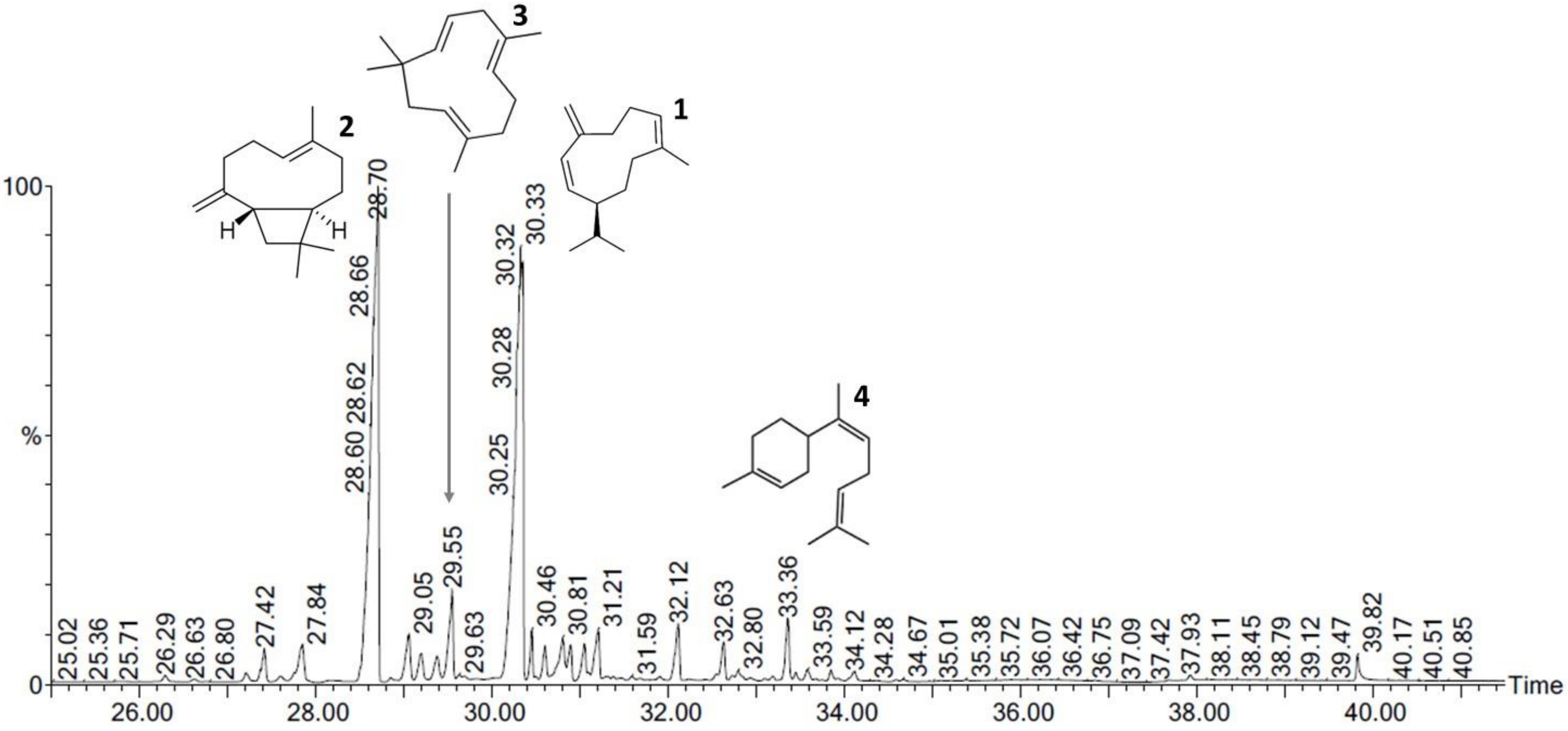

Figure S3. GC-MS Chromatogram of the essential oil extracted from the *Cedrela odorata* dehydrated leaves. Batch 2 collected on September, 2018. Major components: 1= germacrene D, 2 = caryophyllene, 3 = α-caryophyllene, 4 = cis-α-bisabolene.

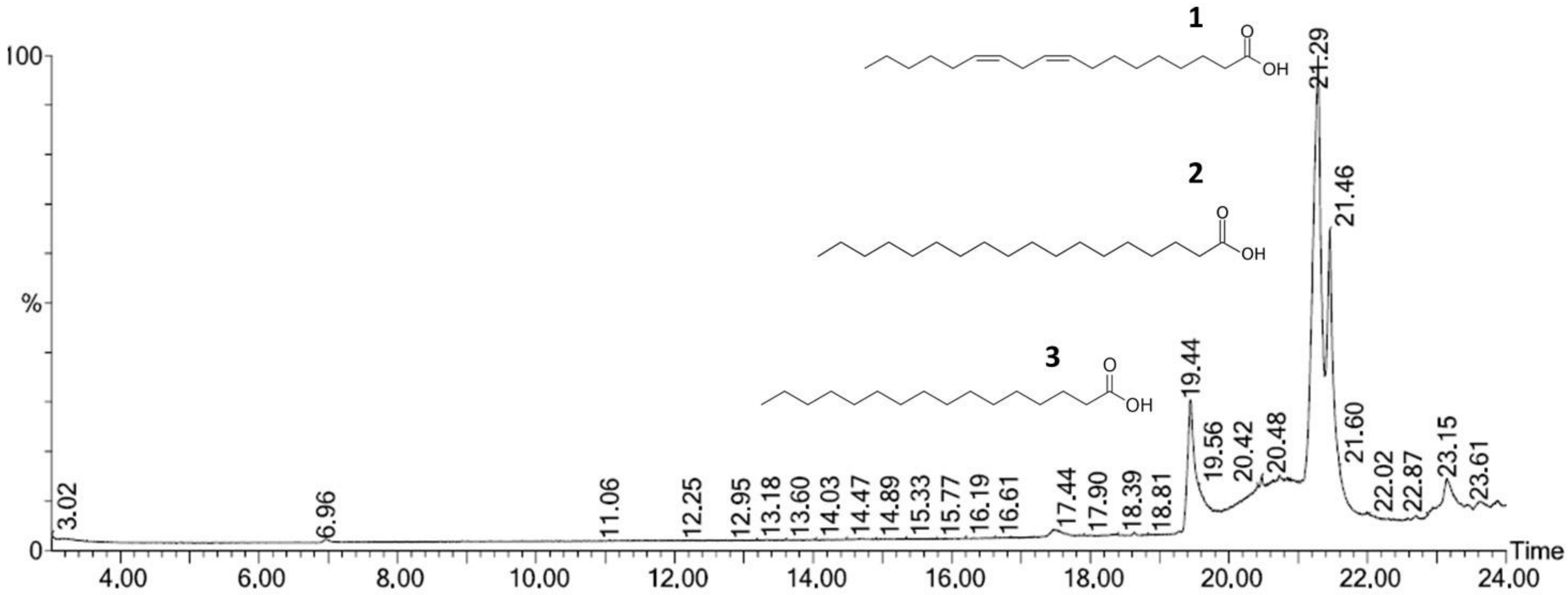

Figure S4. GC-MS chromatogram of commercial Neem essential oil (IBCER S.A. of C.V., Sinaloa, Mexico). Main components: 1= linoleic acid, 2= stearic acid, 3= palmitic acid.

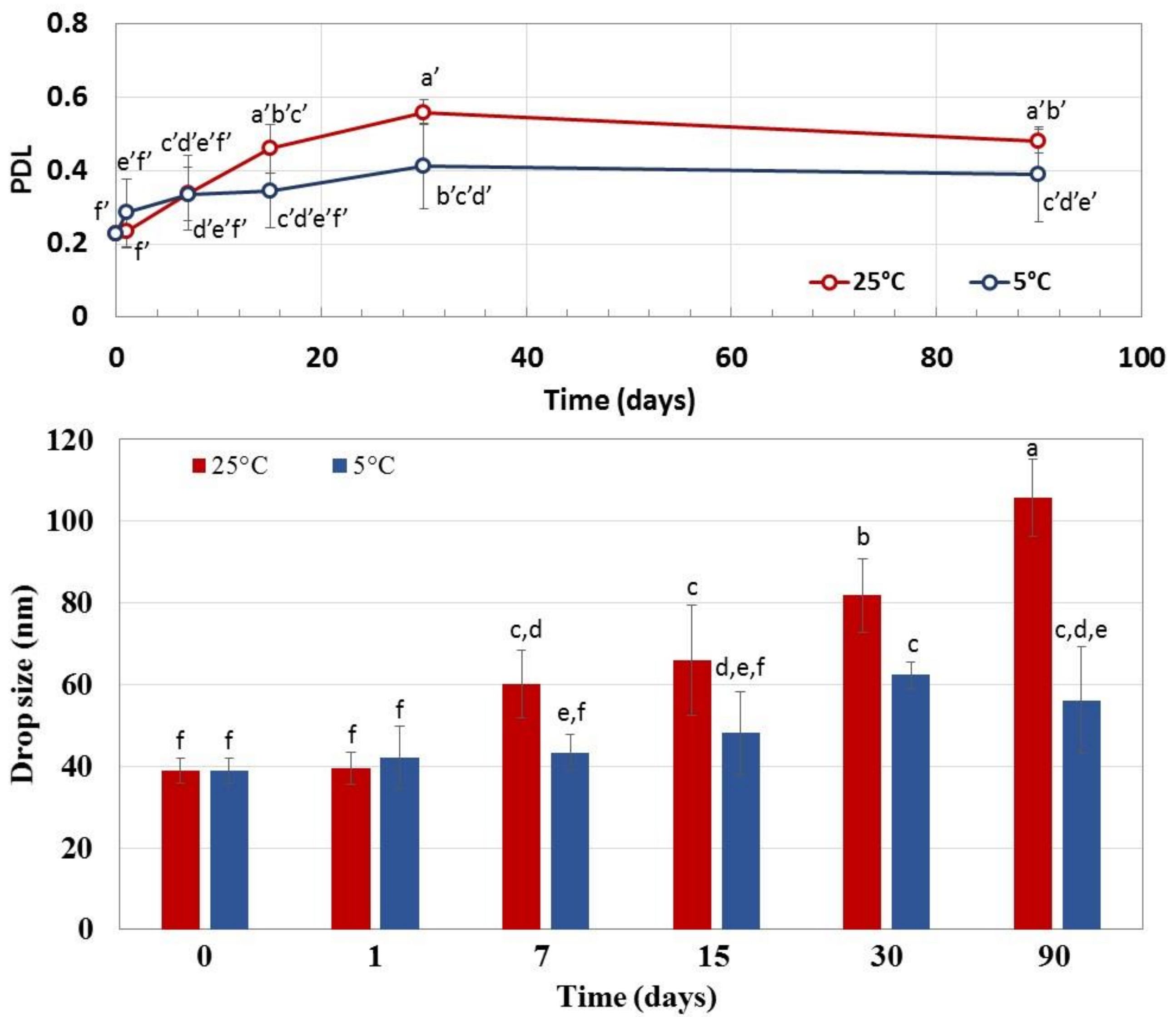

Figure S5. Evolution over time of the droplet size and polydispersity index of a 2.5% nanoemulsion of the essential oil of *Cedrela odorata*. Samples were stored at 25 ± 2 ° C (red) and 5 ± 2 ° C (blue). Two-way ANOVA. Mean values (± SE) that do not share an equal letter (a, b, c, d, e, f) are significantly different according to Tukey's test (α = 0.05).

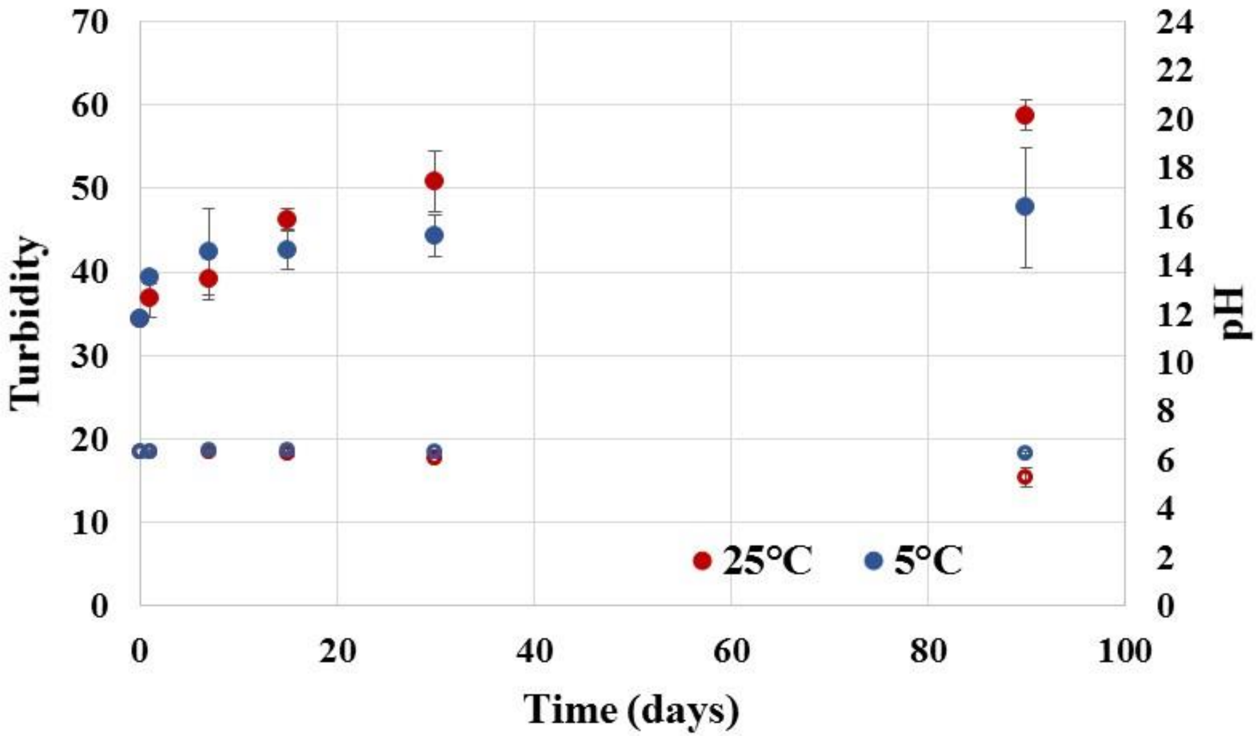

Figure S6. Evolution over time of the turbidity and pH of a 2.5% Cedrela odorata essential oil nanoemulsion. Samples were stored at 25 ± 2 ° C (red) and 5 ± 2 ° C (blue). Two-way ANOVA. Mean values (± SE) that do not share an equal letter (a, b, c, d, e, f,g) are significantly different according to Tukey's test ($\alpha = 0.05$).

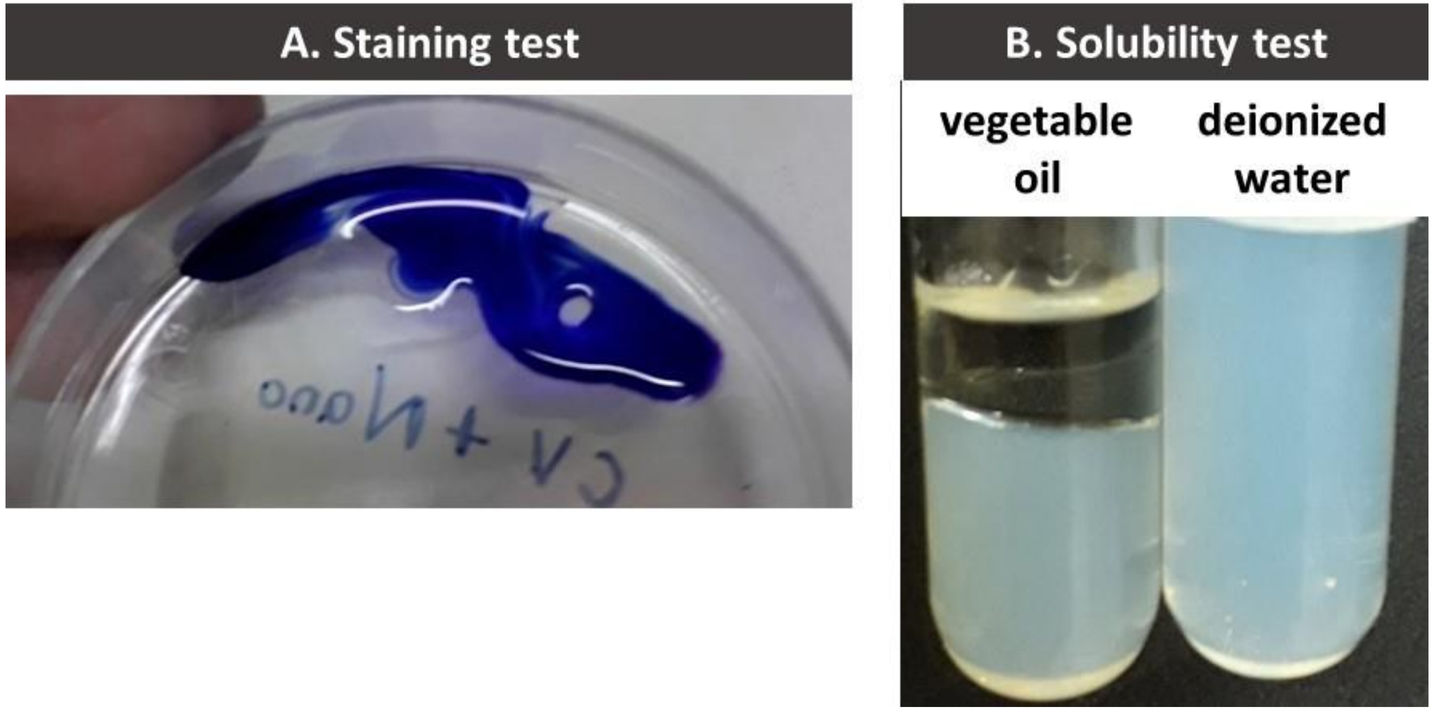

Figure S7. Verification of the oil-in-water type nanoemulsion made with the essential oil of Cedrela odorata and the mixture of tween 80 and Span 80 surfactants. (A) staining test and (B) solubility test

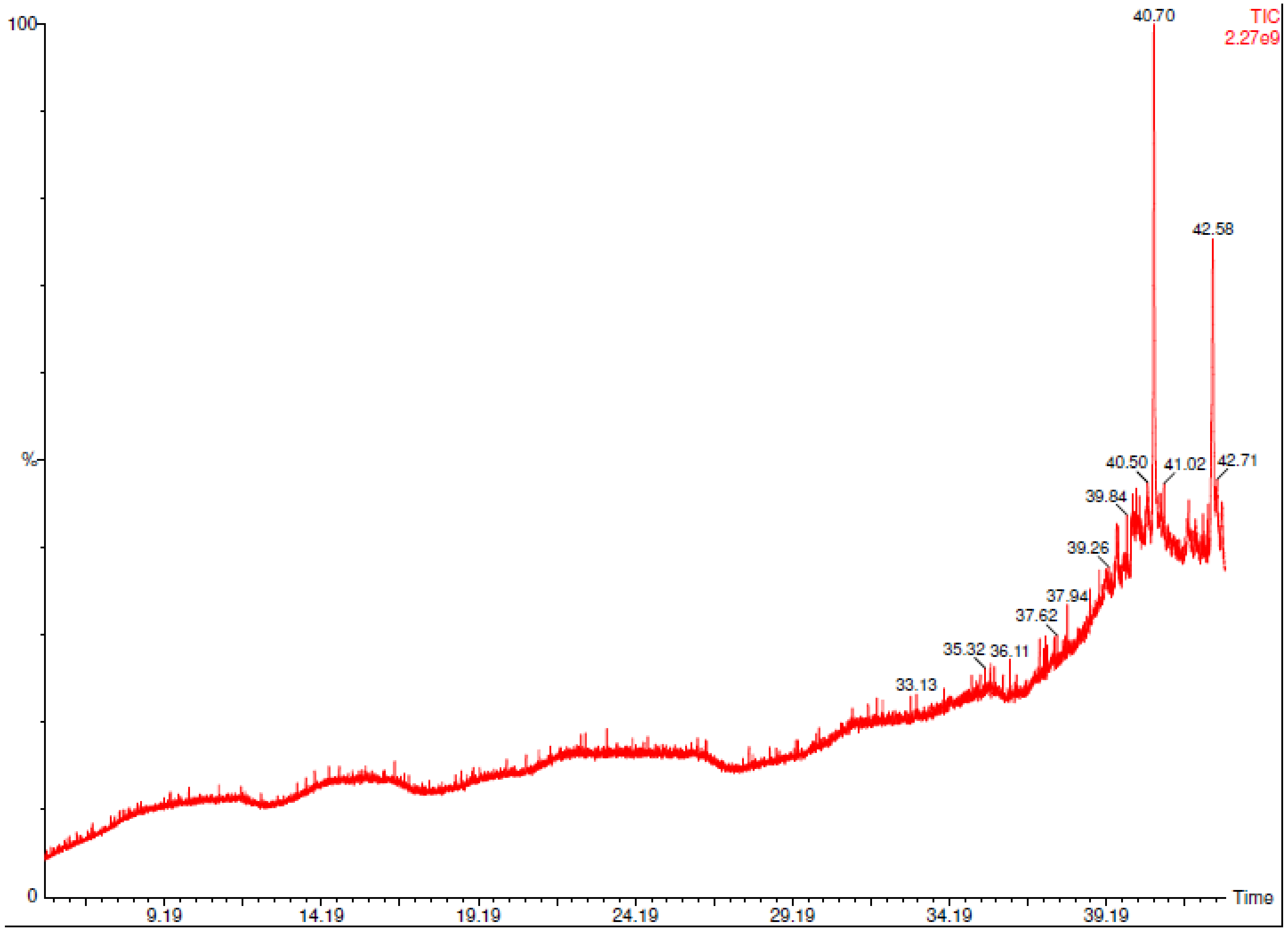

Figure S8. Tween 80 Surfactant Chromatogram

References

1. Sousa-Barbosa, M.; Barbosa-Dias, B.; Santana-Guerra, M.; da Costa-Vieira G.H. Applying plant oils to control fall armyworm (*Spodoptera frugiperda*) in corn AJCS 2018, 12(04):557-562. doi: 10.21475/ajcs.18.12.04.pne822
2. Mascarette Labinas A. and Badiali Crocomo W. Effect of Java grass (Cymbopogon winterianus Jowitt) essential oil on fall armyworm Spodoptera frugiperda (J. E. Smith, 1797) (Lepidoptera, Noctuidae). Acta Scientiarum. 2002, 24(5), 1401-1405
3. Fazolin, M.; Vidal Estrela, J.L.; Monteiro Medeiros, A.F.; Silva, I.M. da; Paiva Gomes, L.; Farias Silva M.S. de. Combining the essential oil of *Piper aduncum* L. with commercial insecticides. Semina: Ciências Agrárias, Londrina, 2016, 37(6), 3903-3914. DOI: 10.5433/1679-0359.2016v37n6p3903
4. Lima, R.K.; Cardoso, M.G.; Moraes, J.C.; Melo, B.A.; Rodrigues V.G., Guimarães P.L. Insecticidal Activity of Long-pepper essential oil (Piper hispidinervum C. DC.) on fall armyworm *Spodoptera frugiperda* (J. E. Smith, 1797) (Lepidoptera: Noctuidae). Acta Amazonica. 2009, 39(2): 377 – 382
5. Cruz, G.S.; Wanderley-Teixeira, V.; Oliveira, J.V.; Lopes, F.S.C.; Barbosa, D.R.S.; Breda, M.O.; Dutra, K.A.; Guedes, C.A.; Navarro, D.M.A.F.; and Teixeira, A.A.C. Sublethal Effects of Essential Oils From *Eucalyptus staigeriana* (Myrtales: Myrtaceae), *Ocimum gratissimum* (Lamiales: Laminaceae), and *Foeniculum vulgare* (Apiales:Apiaceae) on the Biology of *Spodoptera frugiperda* (Lepidoptera: Noctuidae). Journal of Economic Entomology, 109(2), 2016, 660–666. doi: 10.1093/jee/tow005
6. Souza, T.F.; Favero, S.; Conte, C. de Oliveira. Bioactivity of essential oils of eucalyptus species for control of *Spodoptera frugiperda* (JE Smith, 1797) (Lepidoptera: Noctuidae). Revista Brasileira de Agroecologia *Rev. Bras. de Agroecologia. 5(2):157-164 (2010)*
7. Nogueira, T.S.R.; Passos, M.deS.; Nascimento, L.P.S.; Arantes, M.B.deS.; Monteiro, N.O.; Boeno, S.I.daS.; Carvalho, Junior ,A. de; Azevedo, O.deA.; Terra, W.da.S.; Vieira, M.G.C.; Braz-Filho, R.; and Vieira, I.J.C. Chemical Compounds and Biologic Activities: A Review of *Cedrela* Genus. Molecules 2020, 25, 5401; doi:10.3390/molecules25225401.
8. Lourenço, A.M.; Haddi, K.; Ribeiro, B.M.; Corrêia, R.F.T.; Tomé, H.V.V.; Santos-Amaya, O.; Pereira, E.J.G.; Guedes, R.N.C.; Santos G.R.; Oliveira E.E.; and Aguiar, R.W.S. Essential oil of *Siparuna guianensis* as an alternative tool for improved lepidopteran control and resistance management practices. Scientific Reports 2018, 8:7215, DOI:10.1038/s41598-018-25721-0
9. Sombra, K.E.S.; de Aguiar, C.V.S.; de Oliveira, S.J.; Barbosa, M.G.; Zocolo, G.J.; and Pastori, P.L. Potential pesticide of three essential oils against Spodoptera frugiperda (J.E. Smith) (Lepidoptera: Noctuidae). Chilean Journal of Agricultural Research 2020. 80(4): 617-628. doi:10.4067/S0718-58392020000400617
10. Villafañe, E.; Tolosa, D.; Bardón, A.; and Neske A. Toxic Effects of *Citrus aurantium* and *C. limon* Essential Oils on *Spodoptera frugiperda* (Lepidoptera: Noctuidae). *Natural Product Communications* 2011. 6 (9):1389-1392.
11. Duarte, J.L.; Amado, J.R.; Oliveira, A.E.; Cruz, R.A.; Ferreira, A.M.; Souto, R.N., ... & Fernandes, C. P. Evaluation of larvicidal activity of a nanoemulsion of *Rosmarinus officinalis* essential oil. *Revista Brasileira de Farmacognosia*, 2015, 25(2), 189-192.
